# Supplementary material for: Hydrodynamic accumulation of small molecules and ions into cell-sized liposomes against a concentration gradient
Source: Commun Chem. 2020 Mar 9;3:32. doi: 10.1038/s42004-020-0277-2 (PMC9814613; doi:10.1038/s42004-020-0277-2)
Supplement: Supplementary file 1 — Supplementary Information [file 42004_2020_277_MOESM1_ESM.pdf]

Supplementary Information for

Hydrodynamic accumulation of small molecules and ions into cell-sized liposomes against a concentration gradient

Authors: H. Sugiyama[1], T. Osaki[2,3], S. Takeuchi[2,4,*], T. Toyota[1,5*]

[1]Department of Basic Science, Graduate School of Arts and Sciences, The University of Tokyo (3-8-1 Komaba, Meguro, Tokyo 153-8902, JAPAN).

[2]Institute of Industrial Science, The University of Tokyo (4-6-1 Komaba, Meguro, Tokyo 153-8505, JAPAN)

[3]Kanagawa Institute of Industrial Science and Technology (3-2-1 Sakado, Takatsu, Kawasaki, Kanagawa 213-0012, JAPAN)

[4]Department of Mechano-Informatics, Graduate School of Information Science and Technology, The University of Tokyo (7-3-1 Hongo, Bunkyo-ku, Tokyo 113-8656, JAPAN).

[5]Universal Biology Institute, The University of Tokyo (3-8-1 Komaba, Meguro, Tokyo 153-8902, JAPAN).

Correspondence to: takeuchi@iis.u-tokyo.ac.jp (S. T), cttoyota@mail.ecc.u-tokyo.ac.jp (T. T.)

CONTENTS：

Supplementary Discussion:
Plumbing of devices
Design of the microfluidic devices
Evaluation of the performance of the trapping device
Evaluation of the performance of the mixing device
Numerical simulation
Estimation of the amount of FL-ATP required to suppress the fluorescence in the liposome

Supplementary Figures: 1–22

Supplementary Table: 1

Supplementary References

Supplementary Discussion:

Plumbing of devices

The geometrical connection of microfluidic devices, pumps, and valves was shown in Fig. S1a. Importantly, the trapping device was housed by custom-made resin housing (Senshu Scientific Co., Ltd.) to have a reproducible connection between the device and tubes (Supplementary Fig. 1).

We chose a fused silica tube (ϕ = 0.20 mm) sleeved by an ethylene tetrafluoroethylene tube (ϕ= 0.33 mm) for tubes directly connected to the device because the diameter of fused silica is barely affected by a temperature shift and is homogeneous even for a narrow diameter. When a fused silica tube narrower than 0.20 mm was used, the fluid resistance became too high to smoothly relax the pressure, resulting in back flows or unintended flow profiles when the state of the valves and pumps was changed. However, when a wider tube was used, the dead volume became too high to be able to quickly exchange the outer solutions.

Design of the microfluidic devices

The mixing device was designed based on the previous report[1] (Supplementary Fig. 2a).

The rear part of the trapping device was designed to select liposomes with diameters of 11.8–12.2 μm according to our previous report[2]. Thus, only liposomes of the targeted diameter could enter the trapping region. In the device reported herein, occupational trap was accomplished by dynamically changing the fluidic resistance of two pathways before and after trapping liposomes[3,4]. The flow at the entrance of the nest bifurcated into a trap path and a bypass path. When the nest is empty, the fluid resistance of the two paths is equivalent, allowing the liposome to move into the nest. However, the trapped liposome increases the resistance of the trap path, which consequently reduces the amount of flow coming into the nest. As mentioned above, by the size-sorting mechanics at the rear part of the device, liposomes induced into the trapping region are only of the targeted diameter. Therefore, the centroid of the liposomes coming to the entrance after the first trap is deterministically excluded from the trap path for occupied nests. This allows for the occupational trapping of only one liposome in the large nest.

The most important parameters to realize this mechanism in a practical manner are the horizontal (H) and vertical (V) lengths between nests, and the shape of the entrance and exit of each nest. First, the diameter of the nest was fixed to 100 μm, which was designed to be large enough to slow down the flow in the nest. Then, H and V were experimentally optimized to 14 μm and 16 μm, respectively, which were large enough to avoid being clogged by the induced liposomes inside the channel.

There are two key points to consider with regards to the shape of the exit of the nest: the gap width of the contacting part and ensuring that the fluidic resistance is equalized to the bypass path. For the former point, according to our previous study, the gap size is desirable to be 20–25% of the targeted diameter (estimated as 3 μm). The latter point is more complicated because the flow bifurcates after going through the exit, and these bifurcated paths are not completely symmetric. First, considering the structural limitation on the aspect ratio (>0.5) and efficient

alignment of nests in one observation field, the horizontal width of the exit is determined to 8 μm. Second, since the width of the contacting part is much smaller than that of other channels, for the working hypothesis on the experimental optimization, we simplified the fluidic resistance of the trap path to be determined only by the resistance of the exit of the nest. We also assumed that a flow in the microfluidic channel was Hagen-Poiseuille flow. Based on these assumptions and constraints, we experimentally determined the width of the base of the trapezoid to be 10 μm as the exit of the nest.

For the shape of the entrance of the nest, a trapezoidal shape worked better than a rectangular shape, probably because it smoothened the trajectory of the center of mass of liposomes, which have a finite exclusive volume. In addition, as for the alignment of the nests, it is important to place a large number of dummy nests behind the practically observed region because the flow profiles in the vicinity of the front and last column cannot be equivalent. In our design, the nests were placed in 15 columns, and only the first seven columns were used as the region for the observation (Supplementary Fig. 2b).

Evaluation of the performance of the trapping device

First, the streamline in the vicinity of the nest was visualized by tracking fluorescent beads. The green fluorescent microbeads dispersion (25 μL) was diluted to 5 mL by Milli-Q water. The neutral density filters (25% and 50%) equipped to a mercury lamp were opened and the exposure time was set to 5 msec. AVI movie files were analyzed by commercially available software to track the particles (Library Co., Ltd., Flow PTV). For analytical reasons, the flow rate of the outer solution was set to 5 μL/h. The obtained image of the streamline was merged to the bright-field image to identify the location of the nest. As shown in Supplementary Fig. 3, the flow bifurcated at a height of 5–6 μm.

Second, to show that the mechanism described above really works as expected, time courses of the number of nests correctly trapping one liposome (trap number) and incorrectly trapping more than two liposomes (error number) was measured for the two flow rates of the liposome dispersion (5.0 and 6.5 μL h^{-1}) under a fixed flow rate of the outer solution (40 μL h^{-1}) (Supplementary Fig. 4). The liposome dispersion was prepared as explained in the method section. EFM images were taken every minute. After 200 min of the observation, trapped liposomes were flushed at 3000 μL h^{-1}. The procedure was repeated six times for each condition. Note that this experiment was conducted in MANSIONs. Generally, the result well demonstrated the mechanism explained above, and the distribution of the diameter of trapped liposomes was moderately narrow (CV < 10%).

One typical cause of trapping an unintended secondary liposome in an occupied nest is that liposomes with a larger diameter than the targeted value could stochastically slip into the trapping region because of their deformability or interaction with other liposomes in the size-sorting region[2]. These large liposomes clog the bypass path to some extent, and the fluidic resistance of the bypass path becomes the same or higher than that of the trap path. The number of liposomes induced into the trapping region corresponds to the flow rate to induce liposome

dispersion. Thus, the number of liposomes of the untargeted diameter can increase at a high flow rate, resulting in increasing the error number. This tendency is clearly depicted in Supplementary Fig. 4: for 6.5 μL h^{-1} of liposomes dispersion, the trap number increased more quickly and then tended to be unchanged at a higher number (~16) than that of 5.0 μL h^{-1} (~10). The mean diameter of liposomes trapped at 6.5 μL h^{-1} was 12.7 μm, which was larger than that of 5.0 μL h^{-1} (11.7 μm), and its coefficient of variation value at 6.5 μL h^{-1} was also slightly large (10%) compared to that of 5.0 μL h^{-1} (8%). In this sense, a more diluted liposome dispersion was desirable for the accurate trapping, but it resulted in low throughput of the experiments. In our case, the three-fold-diluted liposome dispersion was considered to be the best condition for compatibility with the throughput and accuracy of the trap. The other cause for the increase of error number is that the trapped liposome was too small to dam up the flow into the trap path, or the secondary liposome was small enough to invade the occupied nest. These small liposomes are generated by rupturing the outer membrane of multilamellar liposomes with the target diameter during size sorting or after being trapped. This kind of error was difficult to avoid in the current design. Therefore, it is practically efficient to induce liposomes at a high flow rate (6.5 μL h^{-1}) and to stop induction after a moderate number of liposomes are trapped (typically 10–14).

Finally, to confirm that the small molecules can be rapidly supplied in the nest, GFI_{BG} was measured for both occupied and empty nests during the exchange to the 15 μM uranine/1 mM fructose solution (Supplementary Fig. 5). The liposome dispersion was prepared, and the trapping device was set as explained in the method section. After a moderate number of liposomes were trapped in the nests, the valve position was changed to shut off the flow of the liposome dispersion. After 5 min, the valve position was varied again to exchange the outer solution from a 1 mM fructose solution to a 15 μM uranine/1 mM fructose solution. The process of substitution was recorded as an AVI movie (3 frames sec^{-1}). The GFI of a small rectangular area in a nest was manually measured by the freely available software imageJ (National Institutes of Health, USA) for more than 10 nests for both occupied and empty nests. The time course of the GFI was plotted from 250 sec after the valve state was changed.

Evaluation of the performance of the mixing device

To quantify the performance of the mixing, two indices (flatness (φ) and asymmetry (ε) index), were defined as follows:

$$\varphi(r, v_{tot}) = 1 - \int_0^h \frac{|k(l) - k_{ave}|}{|k_{ave}|} dl \quad (1)$$

$$\varepsilon(r, v_{tot}) = \frac{\left| \int_0^{h/2} k(l)dl - \int_{\frac{h}{2}}^{h} k(l)dl \right|}{|k_{ave}|} \quad (2)$$

where r and v_{tot} are the programmed ratio and sum of the flow rate of the two solutions, respectively, and $k(l)$ is the relative GFI at the position l on the L2 normalized by the GFI of

the unmixed fluorescent solution. The subscript "ave" denotes the average of the relative intensity over the width of the channel. The asymmetry index is important to distinguish a wavy line profile from an asymmetric profile indicating imperfect mixing.

We introduced 200 μM uranine solution and Milli-Q water into the mixing device with different flow rates. First, the ratio of the flow rate of the two solutions (r) was fixed at 0.1, which is the smallest ratio to avoid pulsating flow, and the total flow rate (v_{tot}) was varied in the range of 50–400 μL h^{-1} for every 50 μL h^{-1}. Second, v_{tot} was fixed at 200 μL h^{-1} considering the result of the first test, and r was varied in the range of 0.05 to 0.5. Note that this experiment was also conducted in an automated manner. We took five fluorescence images per minute for each condition. The interval of each condition was 10 min. GFI of the region of interest (L1 and L2 in Supplementary Fig. 6) was measured for these five images. The experiments were repeated three times. The result was shown in Supplementary Fig. 6.

<u>Numerical simulation</u>

To simulate the accumulation of uranine in the trapped liposomes, we set several assumptions to simplify the situation to avoid the overfitting in terms of phenomenological considerations. First, permeation dynamics are described by three kinetics of uranine: permeation from outside to inside (k_{in}) and from inside to outside (k_{out}), and photo-bleaching (k_{bleach}). Second, the fluorescence intensity inside the liposome is proportional to the amount of the intact uranine. Third, no photo-bleached uranine exists in the environment.

Uranine is negatively charged at the current experimental range of pH, and liposomal membrane containing POPG is also negatively charged (Supplementary Fig. S21). Therefore, the Donnan potential can be involved along to the transport of the molecule. Thus, we included the repulsive interactions among the charged molecules confined in the membrane as a correction factor of k_{in} and k_{out}. We heuristically implemented this effect by an exponential function of the whole amount of inner and external charged substances. As explained above, the time course of the amount of uranine in the environment (I_{BG}) was initially the same as the time course of GFI_{BG}, and was then interpolated by the simple arithmetic average of two consecutive intensities:

$$I_{BG}\left(\frac{t(n)+t(n+1)}{2}\right) := \frac{I_{BG}\big(t(n)\big)+I_{BG}\big(t(n+1)\big)}{2} \tag{3}$$

The interpolation was repeated until the time interval ($t(n+1)-t(n)$) became smaller than 0.01. Thus, the amount of intact uranine (I_{int}) and photo-bleached uranine ($I_{bleached}$) was sequentially calculated as follows:

$$I_{int}\big(t(n+1)\big) = I_{int}\big(t(n)\big) + I_{BG} \times \frac{k_{in}}{\exp\left(\frac{I_{tot}}{a}\right)} - I_{int} \times \frac{\mathrm{k_{out}}}{\exp\left(\frac{I_{tot}}{a}\right)} \tag{4}$$

$$I_{bleached}\big(t(n+1)\big) = I_{bleached}\big(t(n)\big) - I_{bleached} \times \frac{\mathrm{k_{out}}}{\exp\left(\frac{I_{tot}}{a}\right)} \tag{5}$$

where I_{tot} is the sum of uranine ($I_{tot} = I_{int} + I_{bleached} + I_{BG}$). Note that I_{int} and $I_{bleached}$ were further calculated at some time points corresponding to when the fluorescence image was taken:

$$I_{int}\big(t(n+1)\big)^{*} = I_{int}\big(t(n+1)\big) \times (1 - k_{bleach}) \tag{6}$$

$$I_{bleached}\big(t(n+1)\big)^{*} = I_{bleached}\big(t(n+1)\big) + I_{int}\big(t(n+1)\big) \times k_{bleach} \tag{7}$$

First, the kinetics of photo-bleaching was estimated experimentally. A lipid film prepared as explained in the method section, was swelled with 5 μM uranine solution. Then the liposome dispersion was diluted 10-fold by the 1 mM fructose solution and immediately put onto a 25-μL specimen with two cover glass slips (thickness ~280 μm). EFM images were taken every 7 sec with the same lens and irradiation conditions used in MANSIONs. The uranine-rich liposomes, the focal planes of which were unchanged for over 10 slices (~70 sec), were manually analyzed with imageJ: the contour was manually fit using the red channel of the image and GFI was measured for the region of interest. The result was shown in Supplementary Fig. 12. According to the result, $k_{bleached}$ was set to 0.004.

Then, other kinetics parameters were fitted. A rapid increase followed by saturation with gradual decrease of the intensity was moderately reproduced when, for example, $k_{in} = 0.05$, $k_{out} = 0.004$, and $a = 5$.

Estimation of the amount of FL-ATP required to suppress the fluorescence in the liposome

According to the pH dependence of FL-ATP (Supplementary Fig. 16), the fluorescence intensity became significantly weak under pH < 4. ATP ionizes in multi steps: the first pKa < 1 and secondary pKa ~1.7, which are reported elsewhere[5]. Let us assume that at least one proton is always deprotonated, and the fluorescence intensity could be smaller than that of the background under the following condition:

$$[\mathrm{H}^{+}]_{lipo} \geq [\mathrm{ATP}]_{lipo} = 10^{-4}(\mathrm{mol}) \tag{8}$$

where $[\mathrm{H}^{+}]_{lipo}$ and $[\mathrm{ATP}]_{lipo}$ are the concentration of proton and ATP, respectively. The volume (V) of a liposome with a diameter of 10 μm is:

$$V = \frac{4}{3}\pi r^{3}|_{r\,=\,5\,\times\,10^{-6}}(\mathrm{m}^{3}) \sim 500\ \times\ 10^{-15}(\mathrm{L}) = 0.5\ (\mathrm{pL}) \tag{9}$$

Thus, the amount of ATP required (R_{ATP}) is calculated as follows:

$$R_{ATP} = [\mathrm{H}^{+}]_{lipo} \times V \sim 0.5 \times 10^{-1} \ \text{(mol)} \tag{10}$$

Here, as a secondary assumption, the percentage of fluids passing one nest is proportional to the ratio of the width of the nest (100 μm) to the whole width of the device (2100 μm). The concentration of FL-ATP was 15 μM, and the flow rate was 40 μL h^{-1}. Therefore, the total amount of FL-ATP introduced into one nest (T_{ATP}) in 5 min is estimated as follows:

$$T_{ATP} = 15 \times 10^{-6} \times \left(40 \times \frac{5}{60}\right) \times \frac{100}{2100} \sim 2.5 \times 10^{-1} \ \text{(mol)} \tag{11}$$

Therefore,

$$\frac{R_{ATP}}{T_{ATP}} \sim 2.0 \times 10^{-5} \tag{12}$$

Thus, if one molecule per 50,000 molecules could be entrapped in the liposome, the considerable suppression of the fluorescence emission of FL-ATP within 5 min could be achieved.

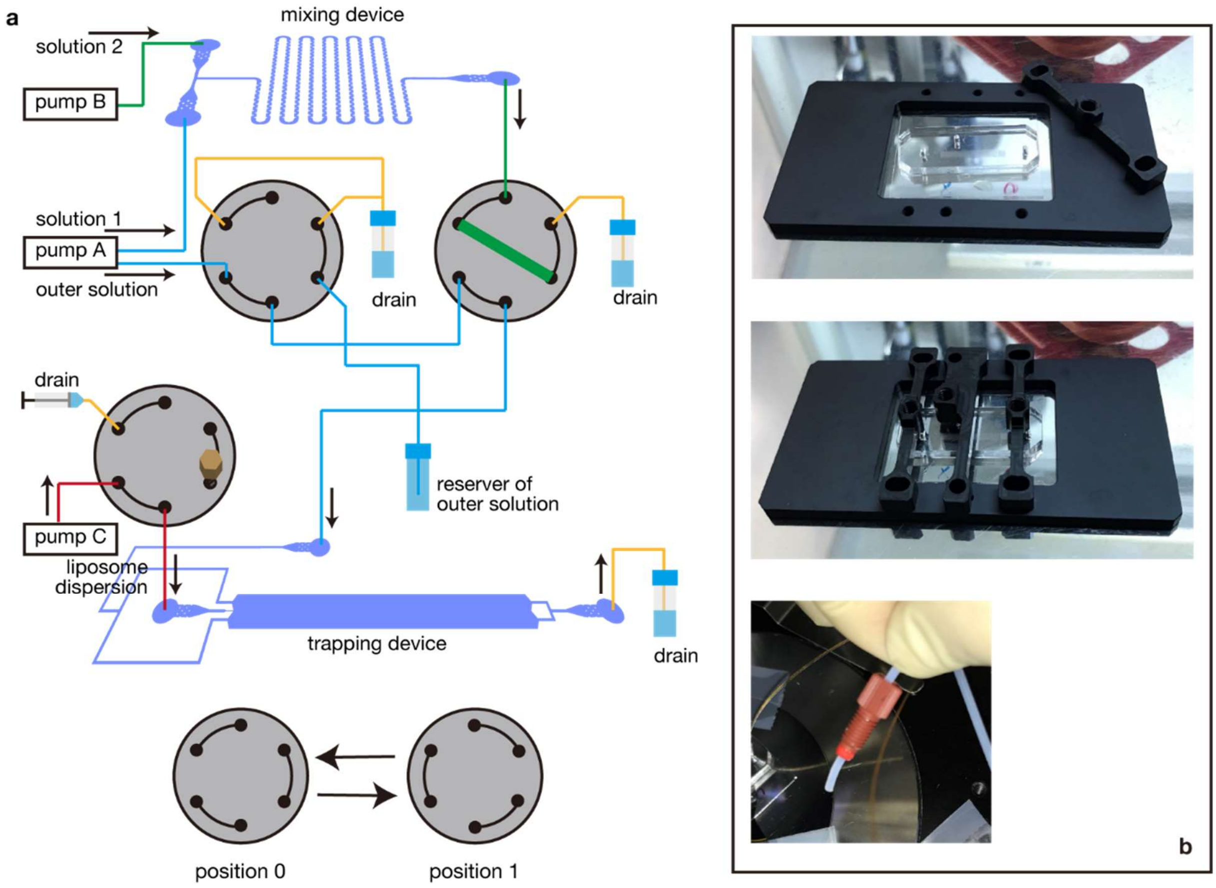

Supplementary Fig. 1 | Conceptual illustration of the experimental set-up of MANSIONs and images of the piping. (a) Detailed illustration for the typical piping structure of the whole system. Blue, green, red, and orange lines denote the pipe for outer solution 1, outer solution 2, liposome dispersion, and drain, respectively. The black curve on the valve shows the flow path in the valve for each position. (b) Photographs of the custom-made housing structure (top and middle) and a tube connected to the observation device (bottom).

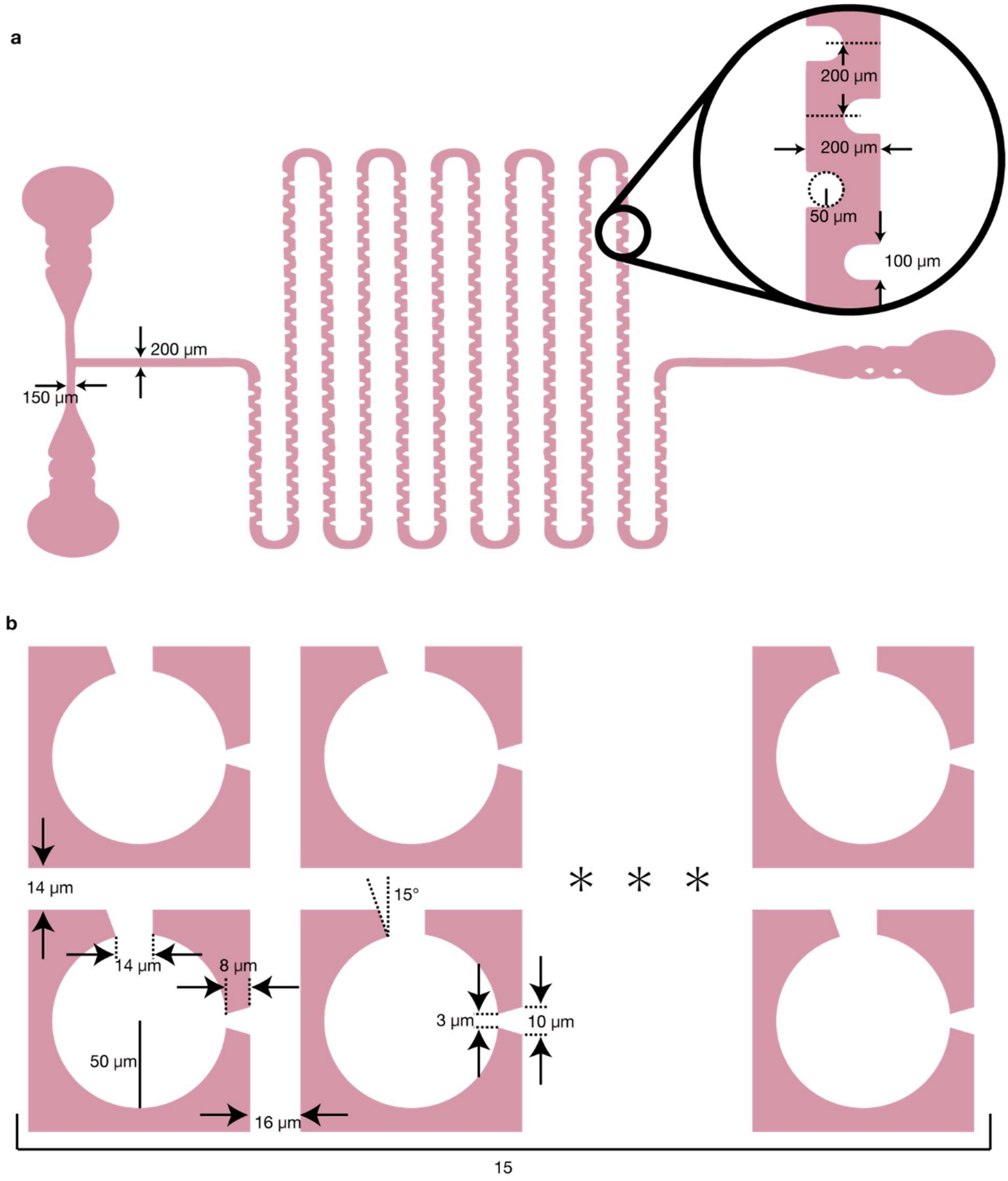

Supplementary Fig. 2 | Important parameters to design microfluidic devices used in MANSIONs. (a) Graphical notation of the design of the mixing device. (b) Graphical notation of the design of the trapping device.

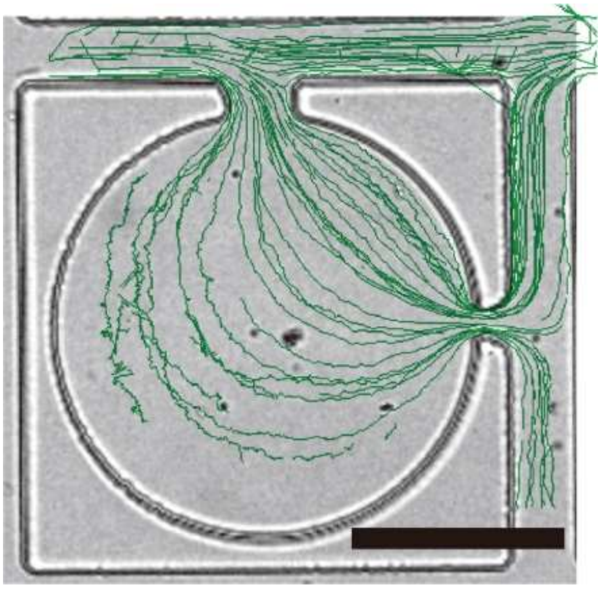

Supplementary Fig. 3 | Streamlines of microbeads flowing in the vicinity of the nest. Digitally generated streamlines are depicted in the bright-field image of the nest. Scale bar: 50 μm.

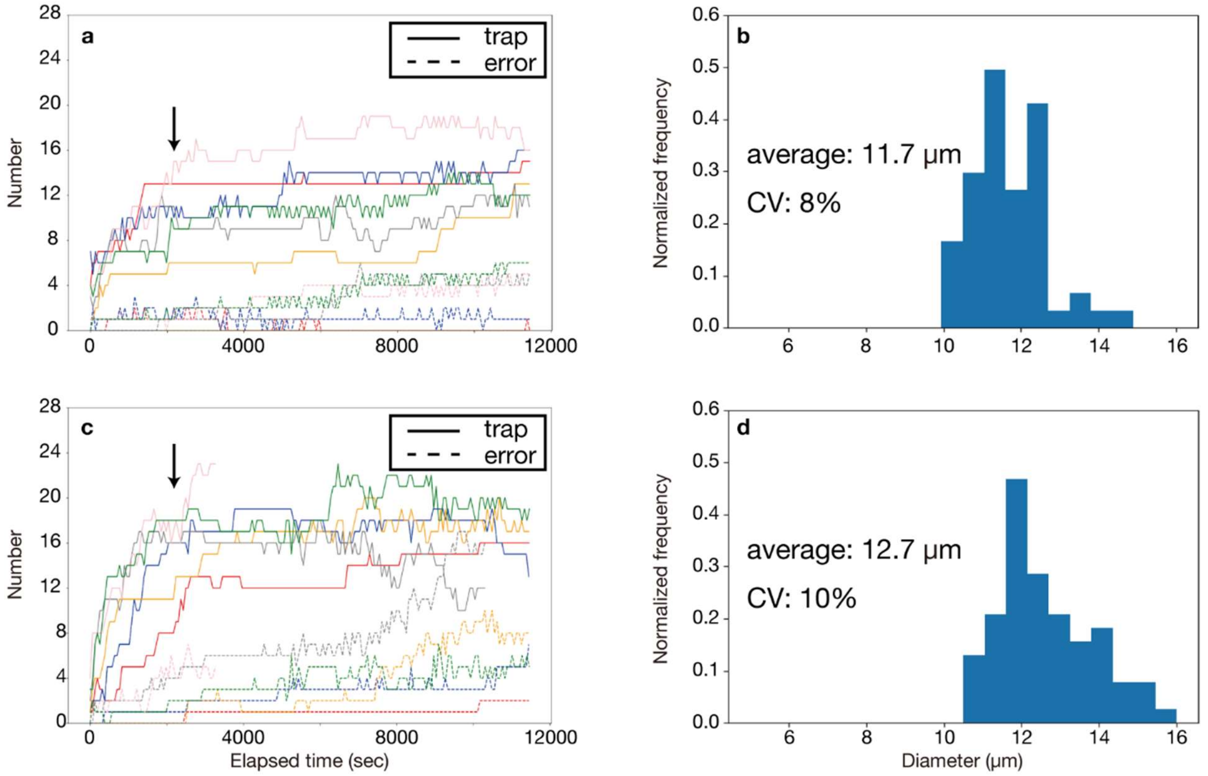

Supplementary Fig. 4 | Verification of trapping mechanism. Time courses of the trap number (solid line) and error number (dashed line), and corresponding histograms at each time point indicated as black arrows. The flow rate of the liposome dispersion was 5.0 µL h^{-1} for (a) and (b), and 6.5 µL h^{-1} for (c) and (d). Plotted lines with the same color were generated from the same experiment.

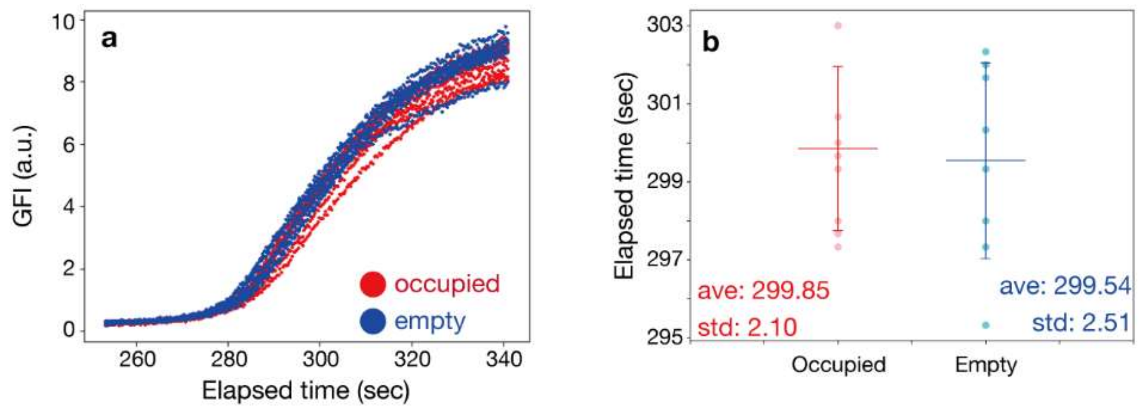

Supplementary Fig. 5 | Comparison of the exchange kinetics of uranine in occupied and empty nests. (a) Time course of GFI of each nest 250 sec after exchange was started. Red and blue dots denote the result of occupied and empty nests, respectively. (b) Time required to achieve half the intensity of the final data point (elapsed time = 340 sec). Pale colored dots (pink and cyan) denote all of the measured data, and intensely colored bars (red and blue, respectively) denote the averaged values (ave) with standard deviations (std).

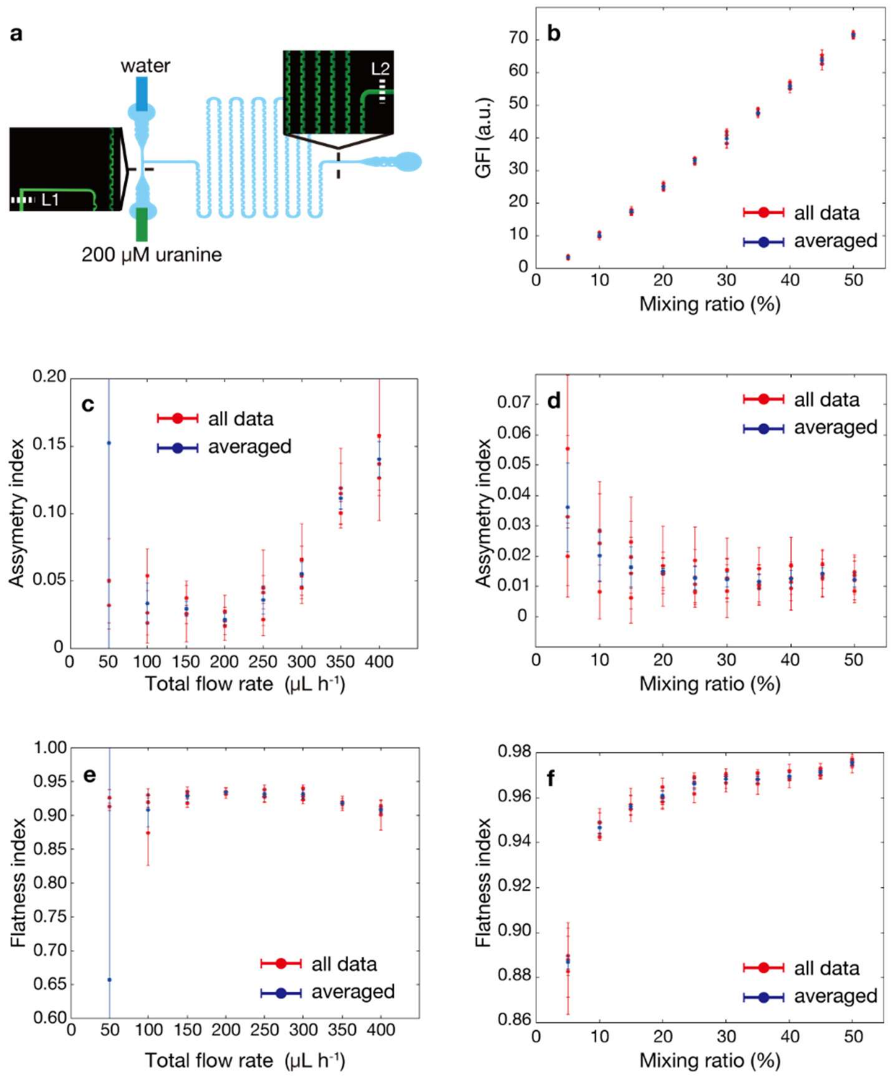

Supplementary Fig. 6 | Evaluation of the performance of the mixing device. (a) Sketch of the experimental settings to evaluate the performance of the mixing device. L1 and L2 represent the lines where the GFI of the original uranine solution and that of uranine solution diluted with water were measured, respectively. (b–f) Plots of the measured GFI (b), asymmetry index (c, d), and flatness index (e, f) to the total flow rate (c, e) and mixing ratio (b, d, and f). Red, blue, and black dots denote the average value of five images taken every minute, the average of three red dots, and data measured for the manually mixed solution, respectively. For (c) and (e), one red dot is outside of the plotted area at 50 µL/h for graphical readability: asymmetric index = 0.38, and flatness index = 0.13. In (b), (d), and (f), the total flow rate was fixed at 200 µL h^{-1}. In (c) and (e), the mixing ratio was fixed at 10%. One red dot and its error bar represent the average and the standard deviation of five consecutive measurements. The experiments were repeated independently three times for each condition, and the average and standard deviation of the three cycles of experiments were plotted as a blue dot with error bar.

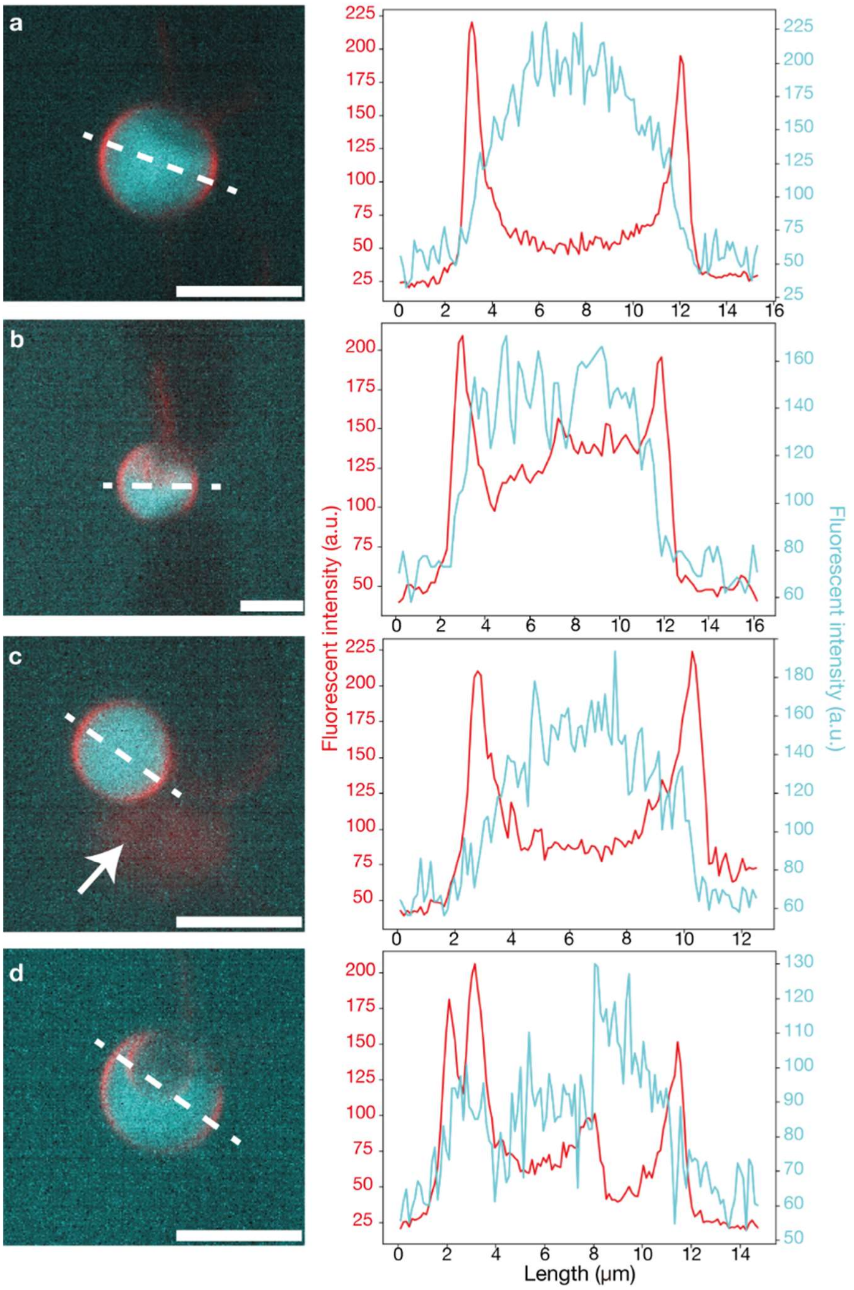

Supplementary Fig. 7 | SDCM images of trapped liposomes exposed to uranine solution under a microfluidic environment. SDCM images in pseudo color (red, 561 nm excitation; cyan, 488 nm excitation) (left) and corresponding line profiles (right). (a–c) Liposomes exposed to a 15 µM uranine/1 mM fructose solution. (d) A liposome at approximately 5 min after starting the washout. The color of the line profiles reflects the color of the SDCM images. The white arrow in (c) is the first liposome (out of focus), and the line profiled liposome is the secondary liposome trapped in the nest considered incorrect trapping. Scale bars: 10 µm.

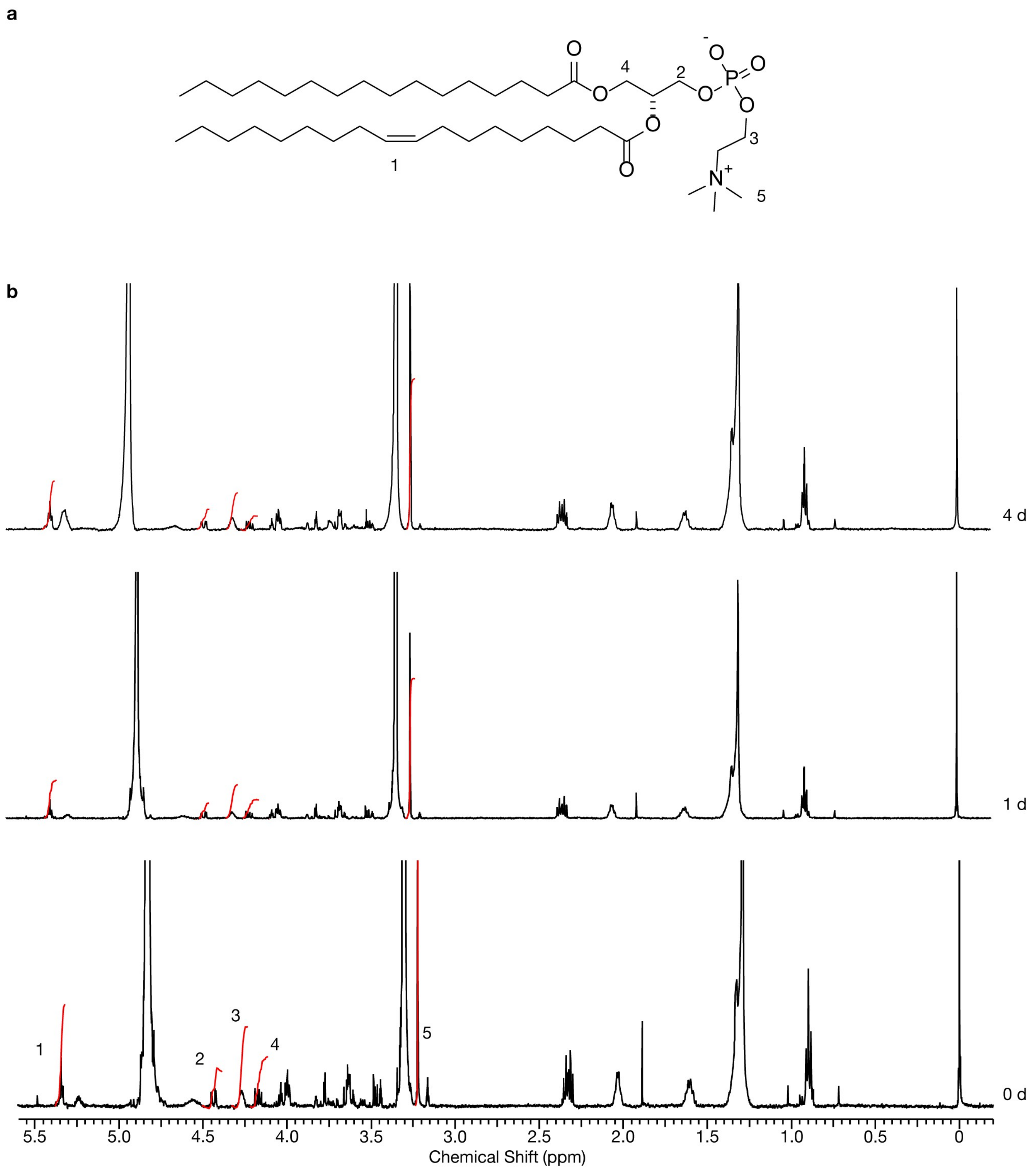

Supplementary Fig. 8 | ^{1}H NMR spectra of the sample of the liposome dispersion (500 MHz, CD_3OD). (a) Molecular structure of POPC, the main component of the liposome used in the experiments. (b) ^{1}H NMR spectra of the liposome dispersion immediately after preparation (0 d), and after 1 and 4 days. Important protons to prevent the oxidation or hydrolysis of phospholipids are denoted by the numbers (1–5) which are common between (a) and (b). The integration values are shown in Supplementary Table 1.

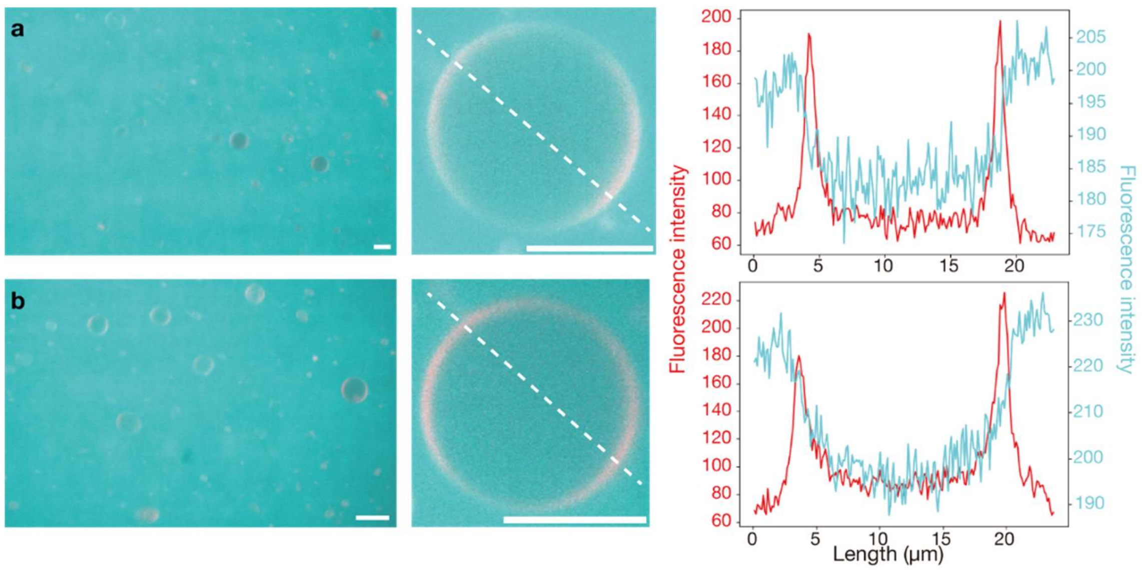

Supplementary Fig. 9 | Liposome dispersion imposed external flow in bulk solutions. SDCM images of low magnification of liposomes (left) and high magnification (middle) of another liposome, and corresponding line profiles of the liposome (denoted as white dashed lines in the SDCM images in the middle) (right) in the liposome dispersion vortexed at 500 rpm for 60 min (a) and vigorously pipetted (5 min) (b). Images are shown in pseudo color (red, 561 nm excitation; cyan, 488 nm excitation). The color of the line profiles reflects the color of SDCM images. Note that high magnification and low magnification SDCM images were taken for the different position of the samples to avoid the photobleaching of uranine. Scale bars: 10 μm.

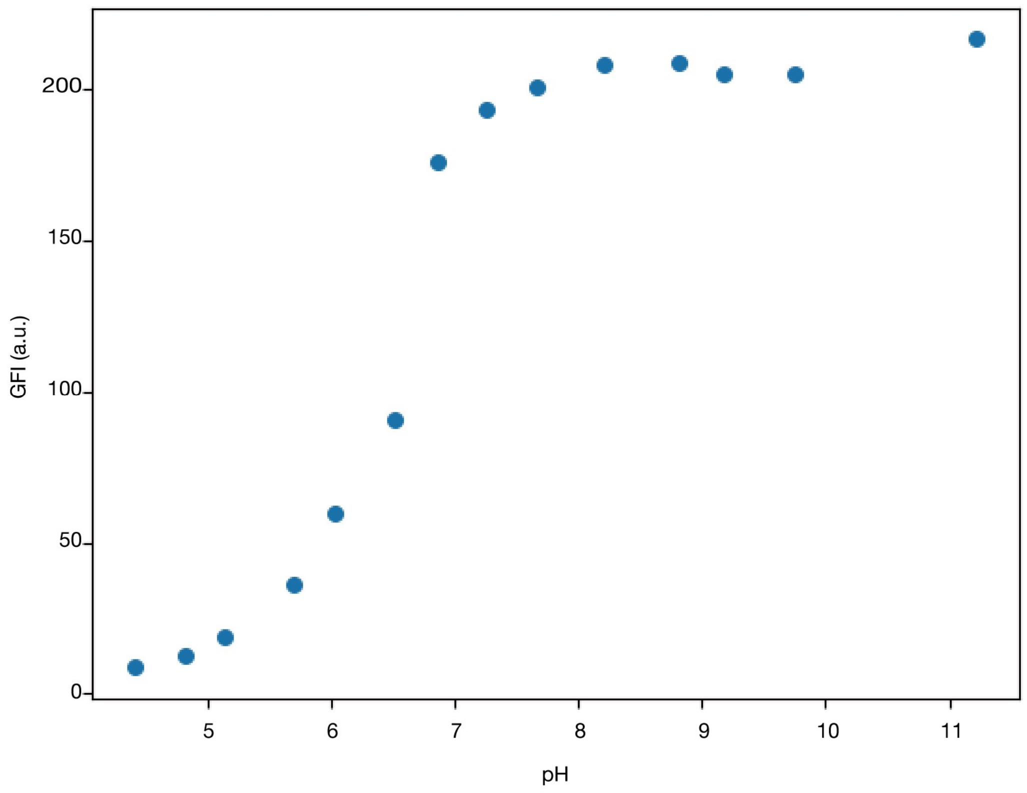

Supplementary Fig. 10| Diagram of the dependency of GFI of the EFM image of uranine solution on pH. The concentration of uranine was fixed at 10 μM, and the exposure time was 25 msec.

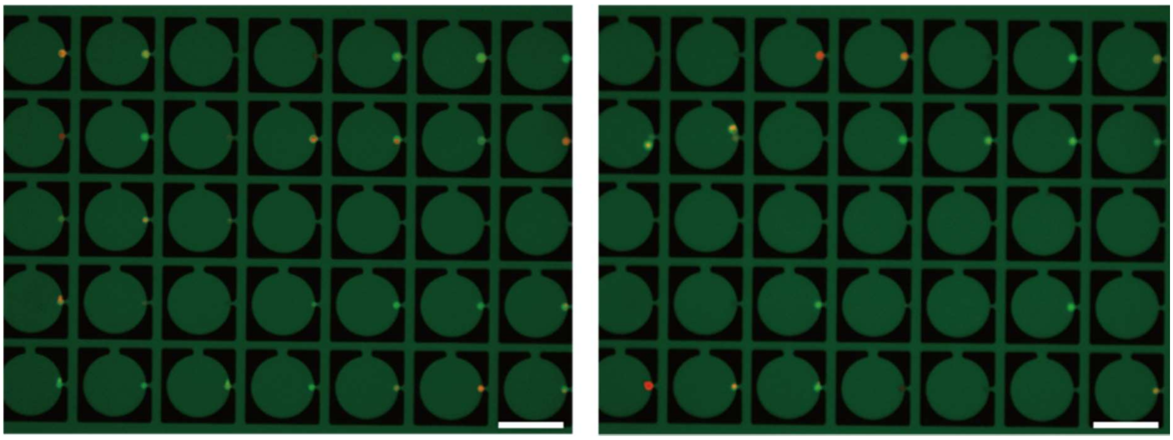

Supplementary Fig. 11 | Liposomes exposed to the buffered uranine solution. Two representative EFM images of the trapping region upon 30 min of exposure to the 5 µM uranine/1 mM fructose/1 mM Tris-HCl solution (arranged to pH 7.87) taken at individual experimental trials. The liposome dispersion was prepared in the swelling of lipid thin film doped with fructose using the buffered solution (5 µM uranine/1 mM Tris-HCl solution (arranged to pH 7.87)). Scale bars: 100 µm.

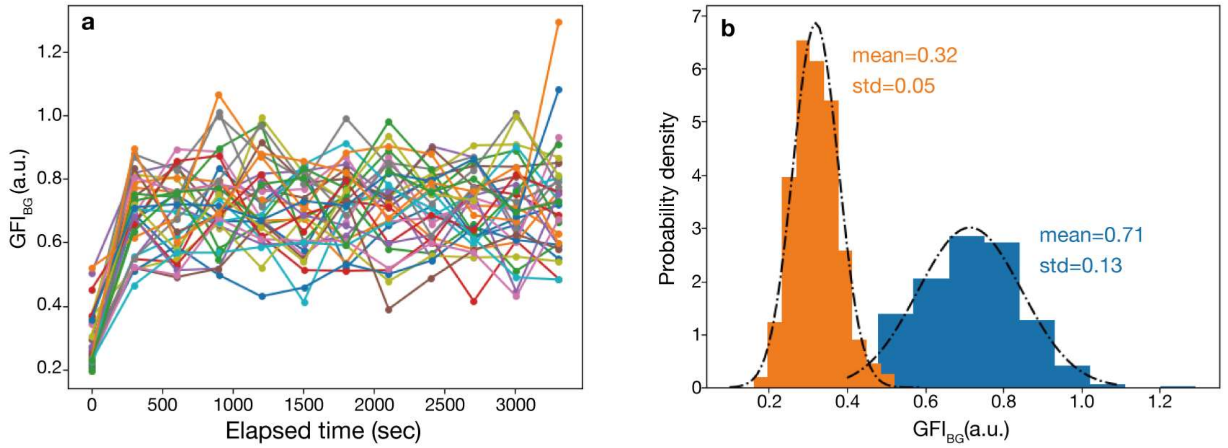

Supplementary Fig. 12 | Assessment of the GFI_{BG} when the diluted uranine solution was introduced to trapped liposomes. (a) Time courses of GFI_{BG} measured for each liposome. (b) Comparison of histograms of GFI_{BG} for 0.5 μM uranine/1 mM fructose solution (blue) and 1 mM fructose solution (orange). Means (mean) and standard deviations (std) of each histogram are shown with the corresponding Gaussian distribution (black dashed lines).

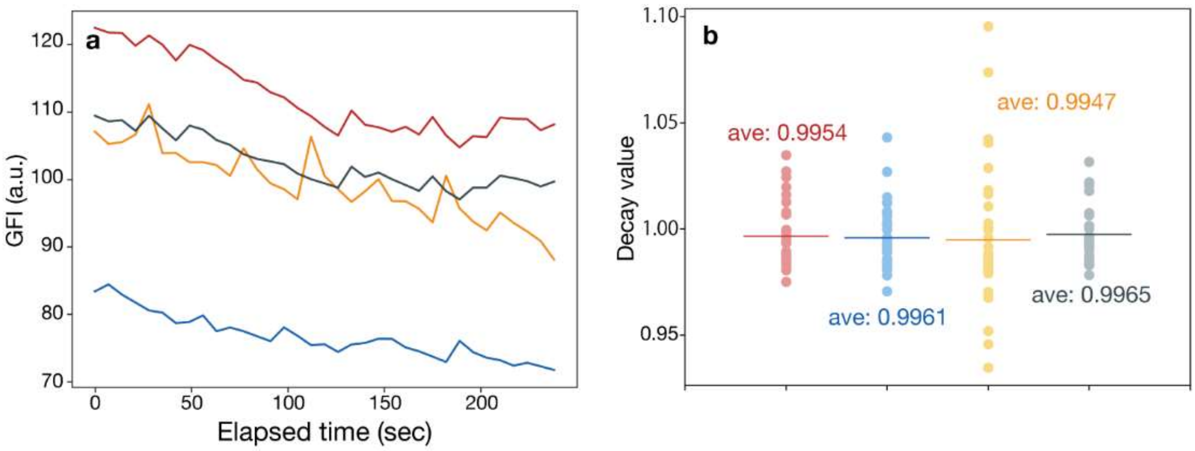

Supplementary Fig. 13 | Photo-bleaching kinetics of uranine solution included in liposomes. (a) Time course of GFI_{lipo}. (b) GFI_{lipo} decay rate, defined as the ratio of GFI_{lipo} to that of just before 7 sec, same as experiments with MANSIONs. Pale colored dots represent all of the measured data, and bars with intense colors denote their average. Colors of each bar are the same in (a) and (b).

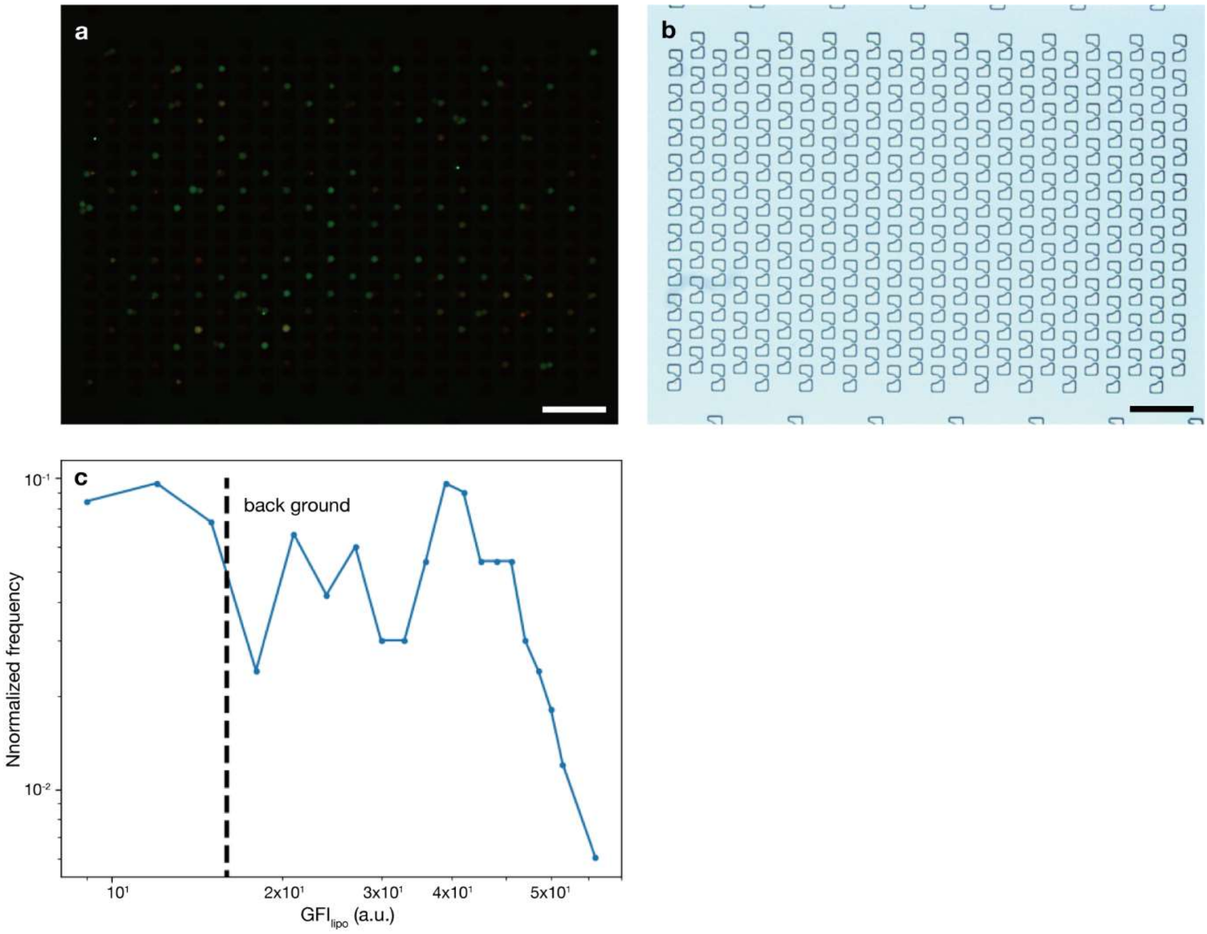

Supplementary Fig. 14 | Liposomes trapped and exposed to uranine solution in the trapping device of another trapping structure. (a) EFM image of liposomes in the trapping region. (b) Bright field microscopy image of trapping region. Scale bars: 100 µm. (c) Double logarithmic plot of the distribution of GFI_{lipo}.in (a). The black dashed line denotes GFI_{BG}.

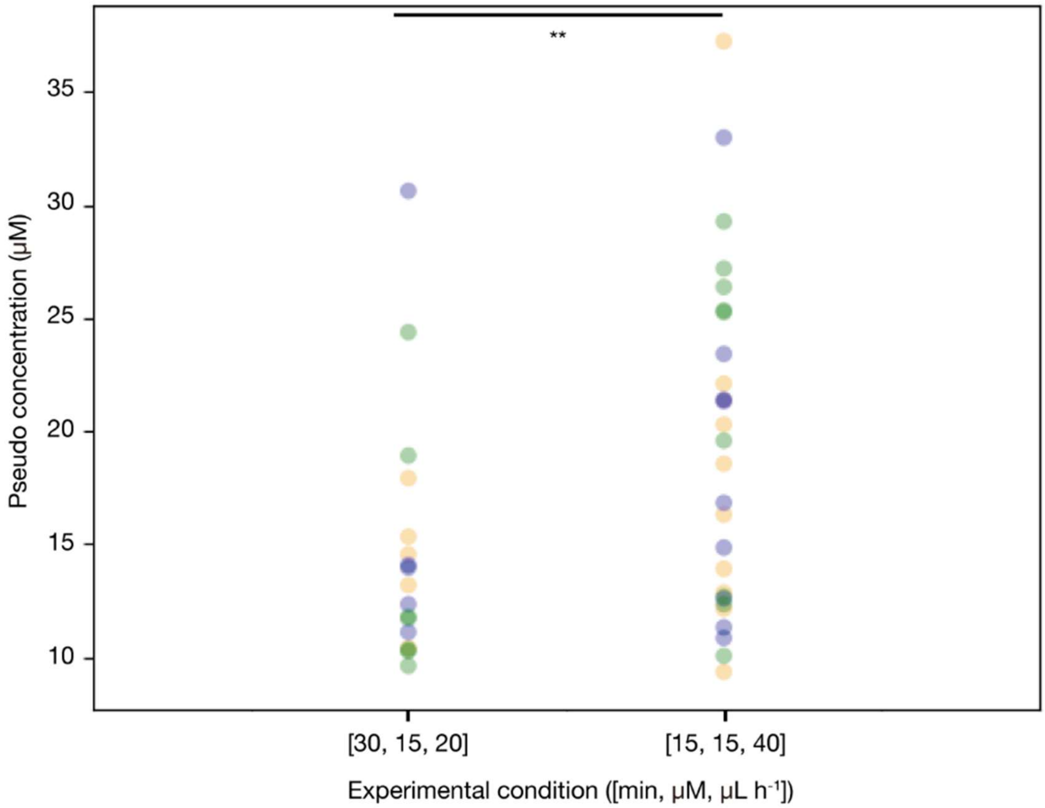

Supplementary Fig. 15 | Distributions of the pseudo concentration of uranine: effect of flow rate during exposure to the uranine solution. Distributions of the pseudo concentration of uranine in liposomes calculated from GFI_{lipo} and GFI_{BG} under different flow rates ([minutes, µM, µL h^{-1}] = [30, 15, 20] and [15, 15, 40]). Data are plotted from three independent measurements for each condition (orange, blue, and green). ** P= 0.033 (< 0.05), two sided Mann-Whitney's U-test.

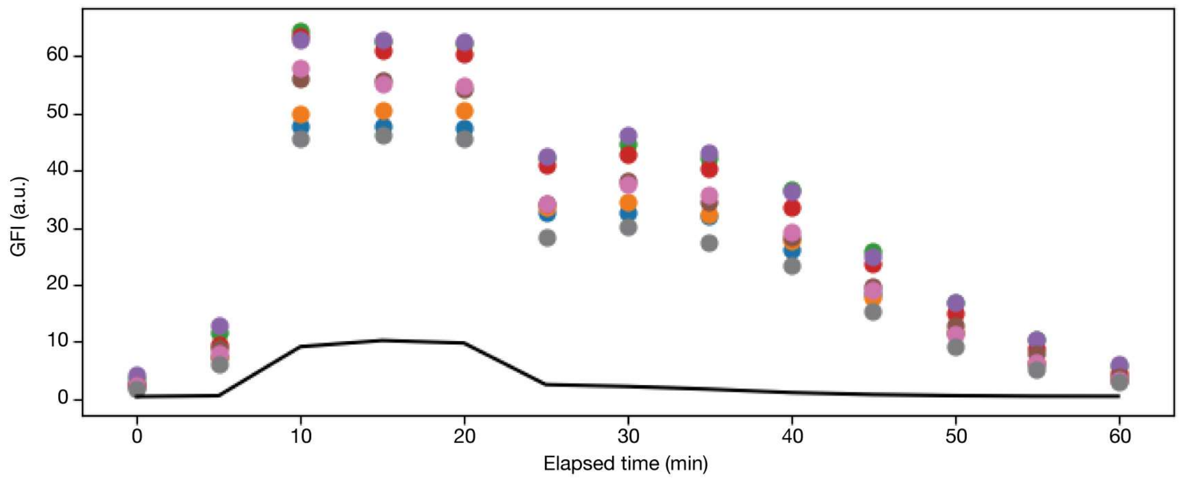

Supplementary Fig. 16 | Liposomes exposed to fluorescein solution (15 µM fluorescein/1 mM fructose). Time course of GFI_{lipo} (colored dots) and GFI_{BG} (black line) upon exposure to fluorescein solution (15 min) and following washout.

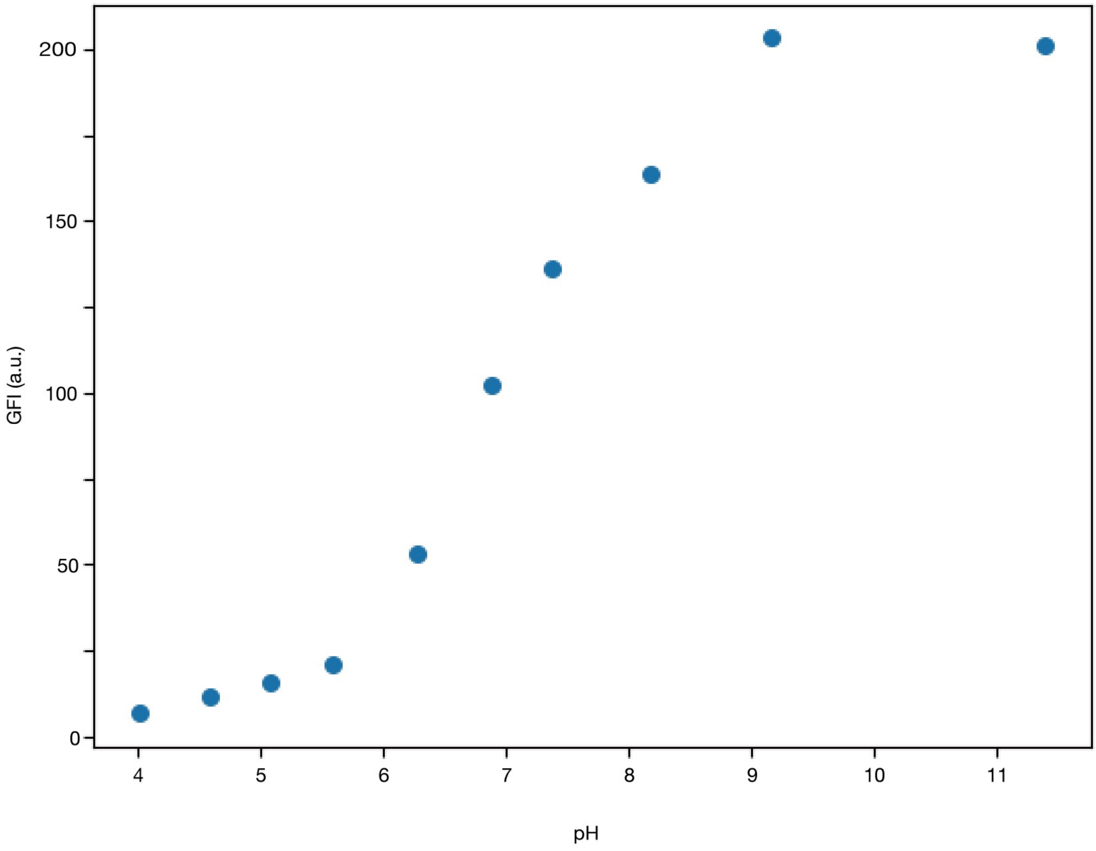

Supplementary Fig. 17 | Diagram of the dependency of GFI of the EFM image of FL-ATP solution on pH. The concentration of FL-ATP was fixed at 5 μM, and the exposure time was 100 msec.

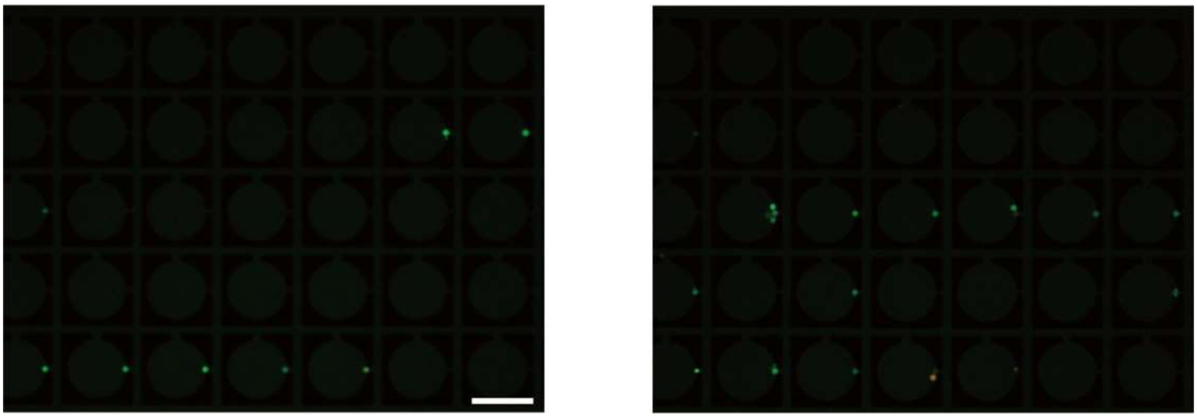

Supplementary Fig. 18 | Liposomes exposed to the uranine under high temperature. Two representative EFM images of the trapping region upon 30 min of exposure to the uranine solution (5 µM uranine/1 mM fructose solution) for liposomes at about 40 °C taken at the individual experimental trials. Scale bars: 100 µm.

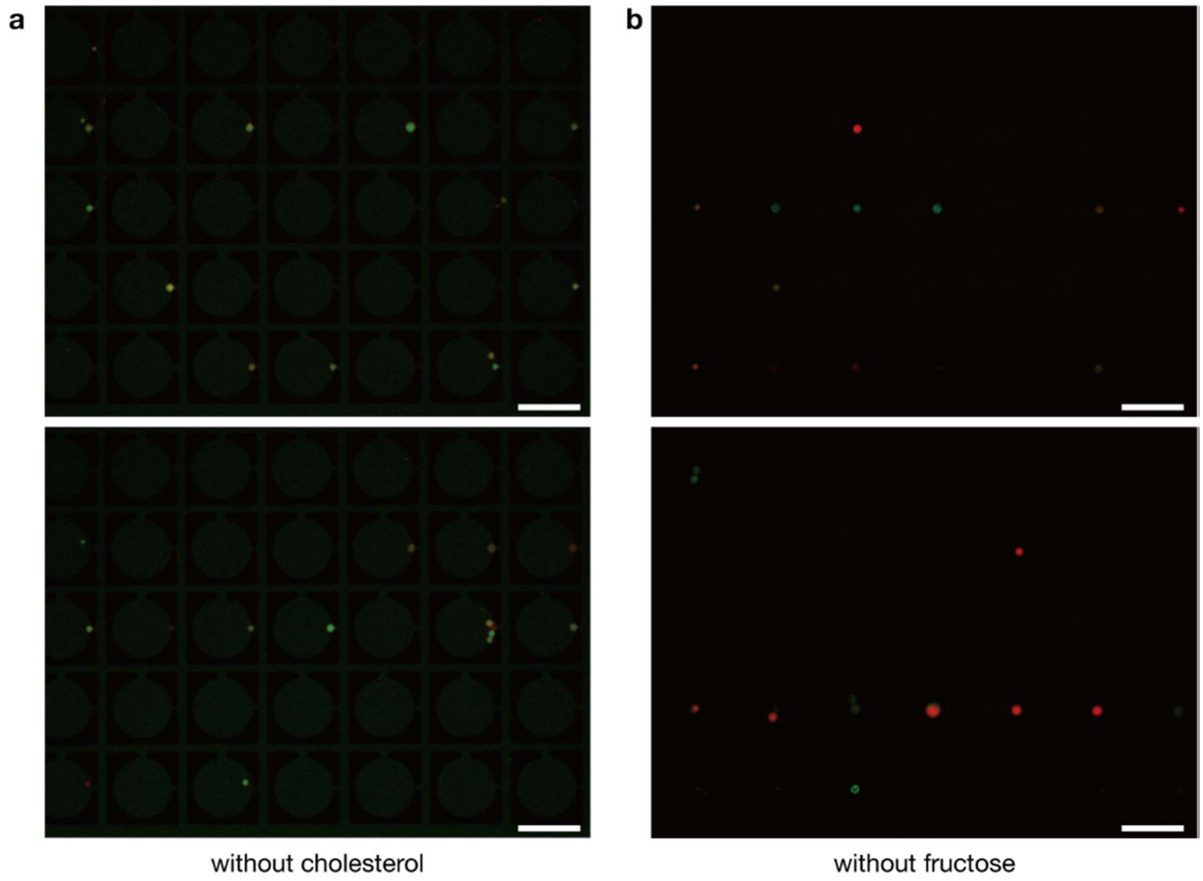

Supplementary Fig. 19 | Exposing liposomes without cholesterol or fructose to the uranine. Two representative EFM images of the trapping region upon 30 min of exposure to the uranine solution for liposomes prepared without cholesterol (a) and without fructose (b) taken at the individual experimental trials. Used uranine solutions were 5 µM uranine/1 mM fructose solution and 5 µM uranine solution respectively. Optical exposure time was 300 msec for (a) and 100 mesc for (b). Scale bars: 100 µm.

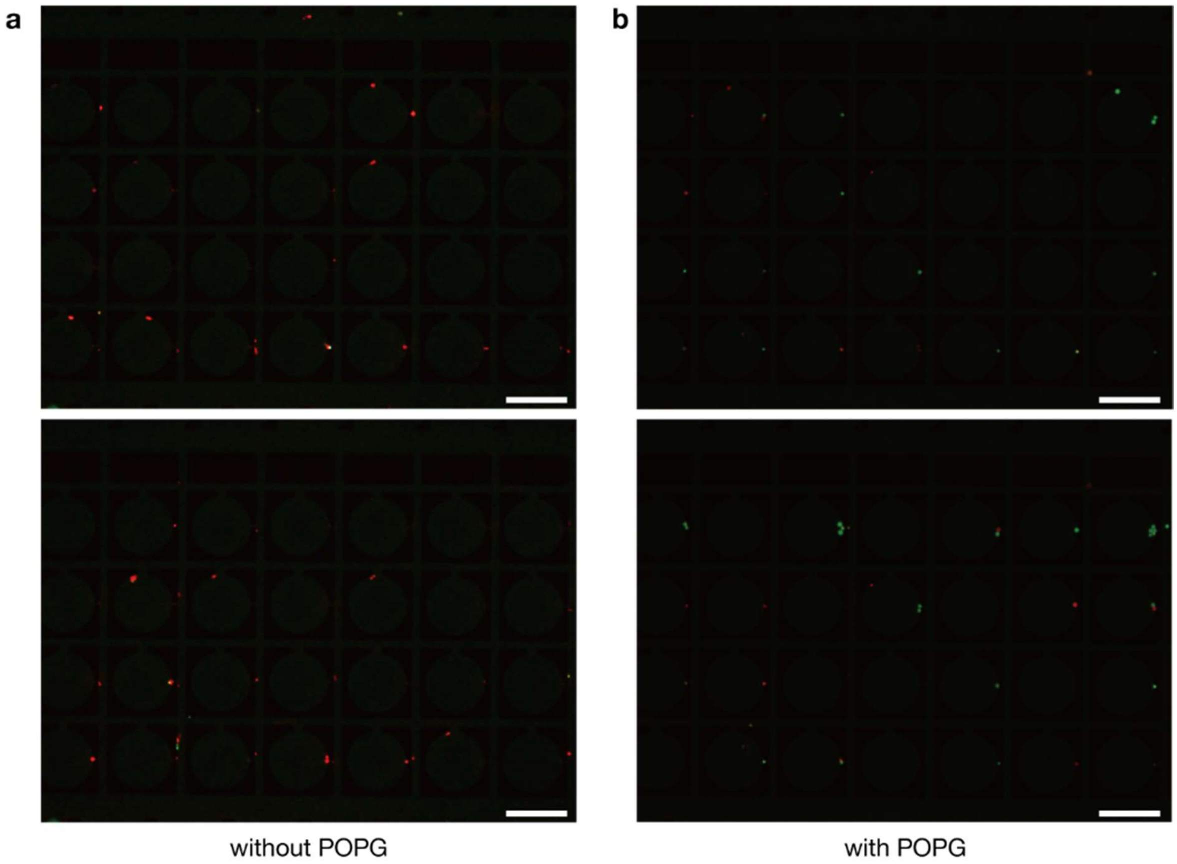

Supplementary Fig. 20 | Exposing liposomes without POPG to the uranine. Two representative EFM images of the trapping region upon 30 min of exposure to the uranine solution (5 µM uranine/1 mM fructose solution) for liposomes prepared without POPG (a) and with POPG (b) taken at the individual experimental trials. Scale bars: 100 µm.

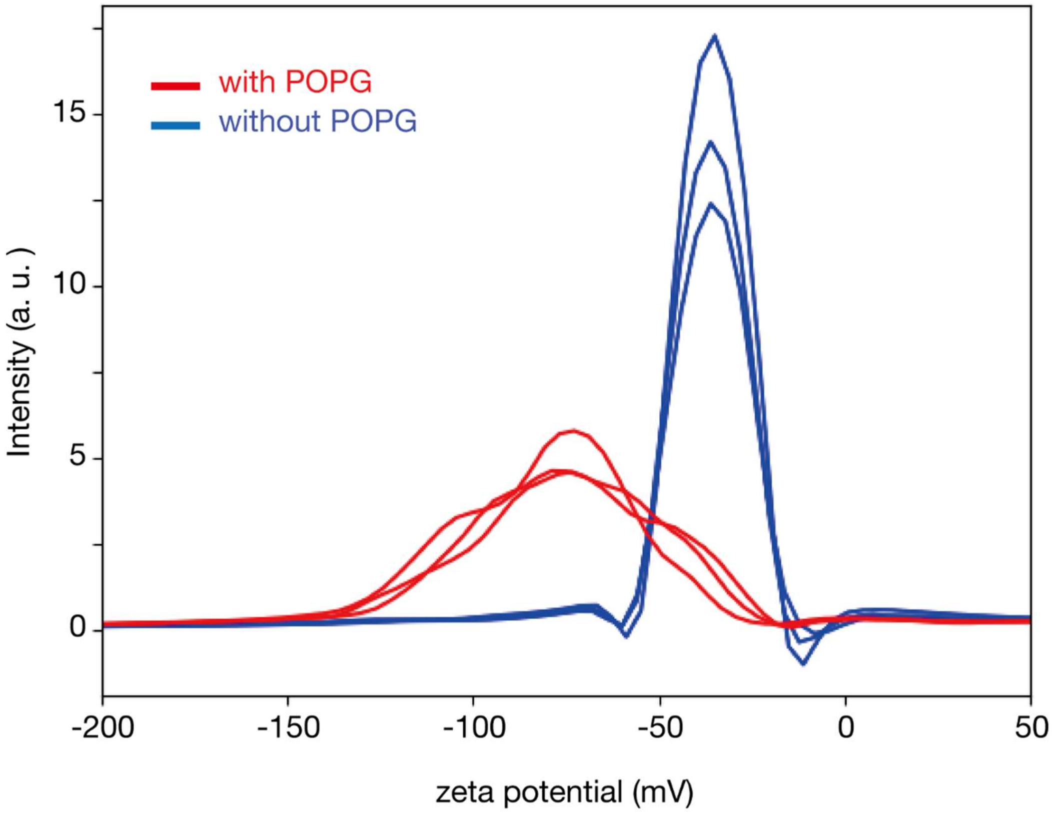

Supplementary Fig. 21 | Effect of POPG on the zeta potential of liposomes. Diagrams of zeta potential for liposomes prepared in presence (red) and absence (blue) of POPG.

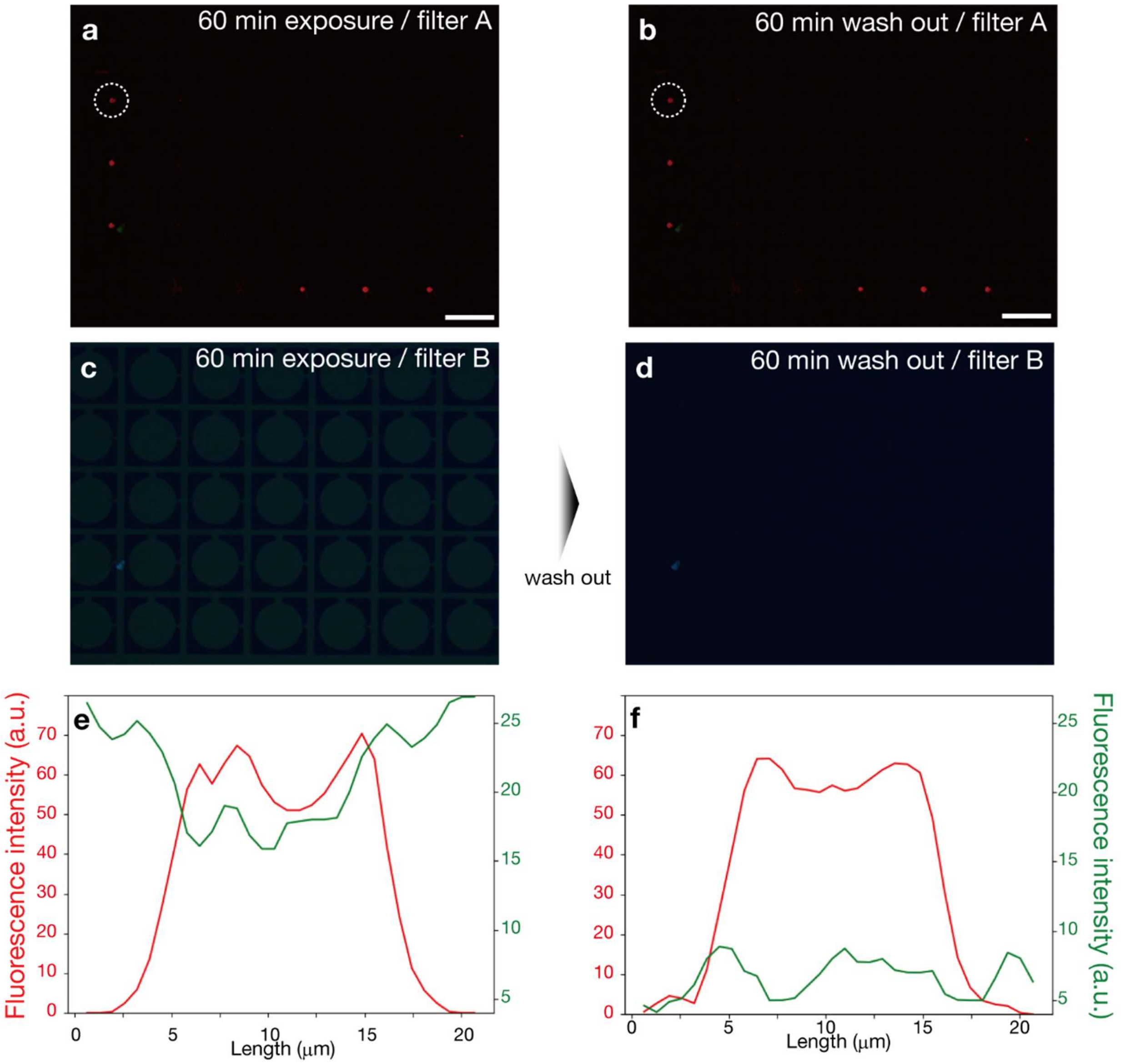

Supplementary Fig. 22 | Liposomes exposed to the BMeS-p-A. Representative EFM images (a–d) and line profiles (e and f) of a representative liposome denoted as white circle after 60 min of exposure to the 15 μM BMeS-p-A/1 mM fructose solution (left), and after 60 min of washout by 1 mM fructose solution (right) The properties of filter A were as follows: excitation, 490–505 nm, 560–580 nm; emission, 515–545 nm, 600–650 nm. The properties of filter B were as follows: excitation, 330–385 nm, emission: >420 nm (long-pass filter). Scale bars: 100 μm.

Supplementary Table 1 | Integration values of proton calculated from the ^1H NMR chart in Supplementary Fig. 8.

Incubation time / days	The place of proton				
	**1**	**2**	**3**	**4**	**5**
0	2.47	0.83	1.94	1.20	9.00
1	2.43	0.96	2.15	1.17	9.00
4	2.13	0.98	2.01	1.02	9.00

Supplementary References:

1 Lee, C. Y. *et al.* Experimental and numerical investigation into mixing efficiency of micromixers with different geometric barriers. *Mater. Sci. Forum.* **505-507**, 391-396, doi:10.4028/www.scientific.net/MSF.505-507.391 (2006).

2 Kazayama, Y., Teshima, T., Osaki, T., Takeuchi, S. & Toyota, T. Integrated microfluidic system for size-based selection and trapping of giant vesicles. *Anal. Chem.* **88**, 1111-1116, doi:10.1021/acs.analchem.5b03772 (2016).

3 Tan, W.-H. & Takeuchi, S. A trap-and-release integrated microfluidic system for dynamic microarray applications. *Proc. Natl. Acad. Sci. U. S. A.* **104**, 1146-1151, doi:10.1073/pnas.0606625104 (2007).

4 Hong, S., Pan, Q. & Lee, L. P. Single-cell level co-culture platform for intercellular communication. *Integrative Biology* **4**, 374-380, doi:10.1039/c2ib00166g (2012).

5 Kaczmarek, P., Szczepanik, W. & Jezowska-Bojczuk, M. Acid-base, coordination and oxidative properties of systems containing ATP, L-histidine and Ni(II) ions. *Dalton Trans*, 3653-3657, doi:10.1039/b508962j (2005).
