# Supplementary material for: Hydrodynamic accumulation of small molecules and ions into cell-sized liposomes against a concentration gradient
Source: Commun Chem. 2020 Mar 9;3:32. doi: 10.1038/s42004-020-0277-2 (PMC9814613; doi:10.1038/s42004-020-0277-2)
Supplement: Supplementary file 2 — Description of Additional Supplementary Files [file 42004_2020_277_MOESM2_ESM.pdf]

Description of Additional Supplementary Files

File Name: Supplementary Movie 1
Description: Flow field in the occupied nest visualized by fluorescent beads. Scale bar: 50 µm.

File Name: Supplementary Movie 2
Description: Liposomes flowing with uranine solution at the size-sorting module. Scale bar: 500 µm.

File Name: Supplementary Movie 3
Description: The change of flow field when the pipe was freed to atmosphere visualized by fluorescence beads. Scale bar: 200 µm.
